# Management of IgA nephropathy and the expanding role of immunomodulation

**DOI:** 10.1016/j.kisu.2026.01.002

**Published:** 2026-07-20

**Authors:** Pietro A. Canetta, Heather N. Reich

**Affiliations:** 1 Department of Medicine, Vagelos College of Physicians and Surgeons, Columbia University, New York, New York, USA; 2 Division of Nephrology, University Health Network, Toronto, Ontario, Canada; 3 Temerty Faculty of Medicine, University of Toronto, Toronto, Ontario, Canada

**Keywords:** biomarker, glomerular filtration rate, IgA nephropathy, immunomodulatory, proteinuria, supportive care

## Abstract

IgA nephropathy (IgAN) requires effective long-term management to avoid kidney failure. Proteinuria and reductions in estimated glomerular filtration rate are important clinical markers of progression, and they are established surrogate efficacy outcomes in clinical trials of new and emerging IgAN therapies. Despite optimized supportive care (including lifestyle modification and renin–angiotensin system inhibition) as a mainstay of IgAN management, many patients will still develop kidney failure, and the concept of “disease modification” refers to interventions that prevent irreversible kidney damage. Disease-modifying therapies may include IgAN-specific and anti-inflammatory strategies, whereas supportive care encompasses interventions that address generic mechanisms of nephron loss and interstitial injury that lead to chronic kidney disease. Targeted therapies, such as Nefecon (targeted-release formulation budesonide) and iptacopan, which address the immune-mediated pathogenic triggers of nephron loss, are important additions. Lifestyle modification, renin–angiotensin system inhibition, sodium-glucose cotransporter-2 inhibitors, and newer agents, such as sparsentan and atrasentan, are also available to manage the generic responses to IgAN-induced nephron loss common to many forms of proteinuric chronic kidney disease. As our understanding of the pathogenic mechanisms underlying disease progression in IgAN has expanded, so has the recognition that IgAN-specific, targeted immunomodulatory therapies are needed, and that these can be used in combination with other agents addressing the more generic causes of nephron loss. These approaches together can help to modify the course of the disease in patients and avoid progression to kidney failure.

When IgA nephropathy (IgAN) was first described in 1968, it was thought to be a relatively benign condition. Since then, it has become evident that a significant proportion of patients with IgAN experience progressive loss of kidney function, which carries a high risk of kidney failure within their lifetime.^1^

**Key Learning Points**

• IgA nephropathy (IgAN) is a chronic progressive disease where “standard of care” has historically involved first addressing the generic responses to IgAN-induced nephron loss. However, these interventions are inadequate to prevent many patients from ultimately progressing to kidney failure.
• The treatment landscape is evolving toward therapies targeting the immunopathogenesis of the disease. It is anticipated that these therapies, in combination with treatments that address the consequences of IgAN-induced nephron loss, will lead to the improved management of IgAN for patients and provide the opportunity to tailor treatments based on risk of progression.

## Markers of Disease Progression in IgAN

Given the variable course of disease progression, interest in predicting which patients are at most risk of progression, as well as in the potential use of biomarkers in the prognosis and management of IgAN, is growing.^2^ Clinical markers of progression risk include histopathologic lesions, decreased glomerular filtration rate, sustained proteinuria, and hypertension,^2^ although changes in these parameters are detected once considerable and often irreversible kidney damage has already occurred.^1^ Genetics and demographic factors, such as race and ethnicity, are unmodifiable risk factors for disease progression.^1^^,^^2^

### Histopathology

Histopathologic scoring of lesions with the Oxford Classification, including mesangial hypercellularity (M), endocapillary hypercellularity (E), segmental glomerulosclerosis (S), tubular atrophy/interstitial fibrosis (T), and crescents (C) (MEST-C score), assists prognosis.^3^^,^^4^ The International IgAN Prediction Tool is a validated prognostic tool that provides individualized risk of a 50% reduction in estimated glomerular filtration rate (eGFR) or end-stage kidney disease (defined as an eGFR <15 ml/min per 1.73 m^2^) up to 7 years from diagnosis and can be used at diagnosis or at a landmark time 1 or 2 years after diagnostic biopsy.^5^^,^^6^ The tool combines histologic kidney findings, including elements of the Oxford score, with demographic and clinical parameters, some of which are nonspecific markers of kidney damage, such as eGFR.^4^, ^5^, ^6^ Currently, this tool cannot be used to select the most appropriate treatment or to determine the impact of a particular treatment regimen, but the data can help inform discussions with patients as treatment options are explored. In the future, this could be used to inform clinical trial design and analysis.

### Proteinuria

The degree of proteinuria in patients with IgAN has been widely studied across cohorts from Europe, Asia, and America and is recognized as the most important modifiable risk factor for progression to kidney failure.^1^^,^^7^, ^8^, ^9^ Proteinuria and time-averaged proteinuria are strong, independent prognostic biomarkers in IgAN. Proteinuria also correlates with histopathologic changes in IgAN, including the presence of mesangial and endocapillary proliferation (i.e., “disease activity”) as well as lesions associated with irreversible injury as a consequence of disease activity, such as segmental glomerulosclerosis and interstitial fibrosis.^10^ Proteinuria levels may also vary within an individual in response to disease-agnostic factors, including exercise, blood pressure, and dietary sodium intake.^7^^,^^11^

Data from the Toronto registry showed that a sustained reduction in proteinuria levels was associated with a reduced rate of eGFR decline and improved renal survival in patients with IgAN.^7^ Similar findings were reported in the Nanjing Glomerulonephritis Registry of Chinese patients with IgAN, where proteinuria levels as low as 0.5 g/d were associated with poorer renal outcomes.^8^ On the basis of these and other similar study findings, the recently updated Kidney Disease: Improving Global Outcomes (KDIGO) guidelines now consider patients with IgAN at risk of progressive loss of kidney function if they have proteinuria ≥0.5 g/d.

Although clinical trials are designed to assess outcomes, not mechanisms, differences in outcomes across different treatment strategies may suggest that the mechanisms by which treatments reduce proteinuria may lead to different degrees of kidney function protection. This may depend on whether the interventions target the primary immunopathogenic mechanisms responsible for IgAN or predominantly have a glomerular hemodynamic effect.^12^, ^13^, ^14^, ^15^ As results of new trials become available, we may also find that there are differences in the relative benefit of each individual approach depending on the mechanism by which immunopathogenesis-targeted therapy acts and on the population studied.^16^^,^^17^ Ultimately, the end goal of interventions is to prevent decline in kidney function; however, the application of proteinuria reduction as a surrogate marker of treatment effect has greatly advanced interest and investment in clinical trials of novel treatments for IgAN.

### Estimated glomerular filtration rate

A reduced eGFR at diagnosis is a well-established predictor of future kidney function decline in IgAN. For example, a study of a Japanese patient population with IgAN found that an eGFR <60 ml/min per 1.73 m^2^ was a significant predictor of progression to kidney failure in patients with IgAN, even with proteinuria <0.5 g/d.^18^ Unfortunately, at the time of diagnosis, many patients already have kidney failure.^7^

A decline of 30% or 40% in eGFR has been studied as a surrogate for kidney failure in clinical trials of treatments in patients with chronic kidney disease (CKD).^19^ However, measuring such a decline can be challenging in patients with IgAN because of the relatively slow progression of the disease, necessitating large, extended trials to detect. Instead, annualized eGFR slope (i.e., the rate of decline in eGFR over time) has been shown to be a viable surrogate end point for kidney disease progression in clinical trials.^19^, ^20^, ^21^ Annualized eGFR slope over a minimum 2-year period has been recognized as an acceptable surrogate allowing full US Food and Drug Administration approval of drugs for IgAN.

Interestingly, a recent meta-analysis evaluating 1-year proteinuria reduction and 1-year eGFR slope as early surrogate end points for disease progression in IgAN showed that the 1-year eGFR slope was an independent predictor of long-term clinical outcomes in IgAN.^22^ Conversely, proteinuria reduction was not an independent predictor of clinical outcomes once the impact of 1-year eGFR slope was accounted for. Indeed, the authors suggest that “sustained effects on eGFR slope are a clear indicator of a disease-modifying treatment effect.”^22^^(page 2731)^ This observation supports the current use of eGFR slope as evidence of efficacy in new drug applications.

Ideally, interventions for IgAN would improve eGFR or at least reduce the annual rate of eGFR decline to that observed in patients without underlying kidney disease. Estimates from the UK National Registry of Rare Kidney Diseases (RaDaR) projected that almost all patients in this cohort are at risk of progression to kidney failure within their expected lifetime unless a rate of eGFR loss ≤1 ml/min per 1.73 m^2^ per year is maintained.^1^

Taken together, these data support that the important treatment targets are to maintain the rate of loss of eGFR at <1 ml/min per 1.73 m^2^ per year, in parallel with achieving maximal reduction of proteinuria. The absolute proteinuria target continues to evolve; however, epidemiologic studies suggest that achieving a level <0.5 g/d, while maintaining eGFR, is a reasonable goal.^1^^,^^8^^,^^23^^,^^24^ It is plausible that even lower levels of proteinuria, down to “normal” levels (e.g., <0.15 g/d), would translate to better long-term outcomes.^25^

## Potential Novel Biomarkers

Our increased understanding of the pathogenesis of IgAN and the development of the multihit hypothesis have expanded the pool of possible biomarker candidates. These include galactose-deficient (Gd) IgA1 and associated Igs (e.g., autoantibodies against Gd-IgA1). They also include those involved in the inflammatory response, such as complement fractions, urinary soluble CD163, or C5b-9.^26^, ^27^, ^28^ Circulating Gd-IgA1 and anti–Gd-IgA1 autoantibodies could potentially be used to monitor the effects of B-cell–targeted or B-cell–activating factor (BAFF)/a proliferation-inducing ligand (APRIL)–targeted therapies, as well as gut-associated lymphoid tissue–directed therapies, such as Nefecon. Conversely, complement fractions could potentially be used to monitor treatment with complement inhibitors, such as iptacopan. Gd-IgA1, prone to self-aggregation, is overproduced in the mucosa-associated lymphoid tissue in IgAN.^29^ Increased serum levels of Gd-IgA1 have been shown to differentiate patients with IgAN from healthy controls and are associated with poorer outcomes.^30^^,^^31^ Furthermore, antibodies against Gd-IgA1 have also been shown to have associations with disease severity.^30^, ^31^, ^32^ Complement activation, most convincingly through the alternative and lectin pathways, contributes to the pathogenesis of IgAN.^33^ Therefore, complement fragments or markers of pathway activation in blood or urine may become useful biomarkers to both reflect disease activity and perhaps guide choice of anticomplement therapy.^30^^,^^33^^,^^34^ Macrophage infiltration is thought to play an important role in IgAN, and the number of macrophages observed in the glomerulus is correlated to the level of hematuria.^35^ Urinary soluble CD163 is shed by macrophages and is correlated with IgAN severity; interim results from the Study of the Safety and Activity of Sparsentan for the Treatment of Incident Patients With Immunoglobulin A Nephropathy (SPARTAN) trial demonstrated that urinary soluble CD163 levels were reduced with sparsentan treatment, although the number of participants was small (Komers R, Cheung CK, Moody S, et al. Sparsentan [SPAR] as first-line treatment of incident patients with IgA nephropathy [IgAN]: interim analysis of the SPARTAN trial [poster]. Presented at: World Congress of Nephrology. February 6–9, 2025; New Delhi, India. Poster WCN25-AB-1884).^36^ These biomarkers are yet to be validated for clinical use, and it is hoped that, in the future, they may be used to predict clinical outcomes with drug interventions and to tailor therapy.

Novel biomarkers are discussed in more detail in the article titled “The expanding role of biomarkers in the management of IgA nephropathy” by Jain and Rizk^37^ in this supplement.

## Management of IgAN: Evidence Supporting Available Treatments

The goal of treatment in IgAN is to preserve kidney function and prevent progression to kidney failure within the patient’s lifetime.

Following the acceptance of proteinuria as an early surrogate marker of kidney disease progression for accelerated drug approvals, scientific progress in developing new approaches to the treatment of IgAN has been rapid. Recommendations regarding the order, sequencing, and selection of the best treatment for individual patients are expected to evolve rapidly as phase 3 trial data on eGFR effects are published. Even the tenet of only offering disease-modifying treatment to patients *after* a trial of supportive care will need to be revisited as safer targeted therapies come to market. Understanding that this is an area of rapid evolution, the evidence supporting the available treatments in the 2 pillars of treatment—supportive and targeted immunomodulatory therapies—are reviewed below.

### Supportive care

Optimized supportive care has historically been the mainstay of IgAN management and remains essential in updated guidelines.^24^ Lifestyle modifications include weight control, endurance exercise, dietary sodium restriction, and cessation of smoking and vaping.^24^ Blood pressure should be controlled, and these guidelines suggest a target blood pressure of ≤120/70 mm Hg. Reduction of glomerular hyperfiltration and the impact of proteinuria using renin–angiotensin system inhibitors (RASis) remains a key recommendation for all patients.^24^ Finally, patients with IgAN should also have their cardiovascular risk assessed and relevant interventions initiated as required in accordance with their GFR and risk profile.^24^

There are patients with IgAN who achieve target proteinuria reduction with supportive care alone. For example, almost a third of patients (106 of 309) with IgAN who received optimized supportive care in the run-in phase of the Supportive Versus Immunosuppressive Therapy for the Treatment of Progressive IgAN (STOP-IgAN) trial went on to experience a decrease in proteinuria (<0.75 g/d), which subsequently meant they were ineligible to remain in the study.^16^ However, despite optimized supportive care, patients with IgAN experience kidney function loss over time.^17^ The 10-year follow-up results from the STOP-IgAN study revealed that patients randomized to receive supportive care experienced an average eGFR loss of ≈2.5 ml/min per 1.73 m^2^ per year. Given that the average age of patients included in the trial was 46 years, this strongly suggests a high proportion of these patients will reach kidney failure during their lifetime.^1^^,^^16^^,^^17^ Overall, although supportive care is an essential initial step, it does not sufficiently maintain a patient’s kidney function in the long-term.

Looking specifically at RASis, small-scale studies have suggested they decrease proteinuria in patients with IgAN.^38^, ^39^, ^40^, ^41^, ^42^ When these studies are analyzed in aggregate, such as through systematic review, it is difficult to establish the impact on kidney failure outcomes. However, this does not necessarily reflect a disconnect between proteinuria reduction and GFR loss. Available studies assessing RASis specifically in IgAN are small in size and short in duration, yielding low levels of certainty regarding their conclusions.^41^ Kidney end points differed from those evaluated in more contemporary studies of similar duration; end points included primarily halving of eGFR or kidney failure, which are unlikely to occur during the study period, particularly in lower-risk populations. Data support pleiotropic effects of RASis on glomerular and tubular injury related to their role moderating the hemodynamic and nonhemodynamic effects of angiotensin II on the kidneys.^41^^,^^43^ However, a lack of impact on kidney failure in a short time frame may also reflect the fact that the degree of impact of RASis on mitigating disease injury is relatively small compared with an intervention that impacts core mechanisms of disease pathogenesis. Larger trials of RASis, conducted over longer periods and designed to evaluate more contemporary end points, are unlikely to be performed, as these medications are standard elements of care in patients with CKD. In general, RASis are beneficial supportive interventions for IgAN, with the benefits outweighing the potential adverse effects.^41^

### Sodium-glucose cotransporter-2 inhibitors

Following investigation of sodium-glucose cotransporter-2 inhibitors (SGLT2is) in patients with type 2 diabetes, they were found to slow the rate of decline of eGFR and reduce albuminuria independent of their glucose-lowering effects. This was thought to relate to their impact on reducing intraglomerular pressure and hyperfiltration injury, prompting the study of these medications in patients with nondiabetic CKD.^44^^,^^45^

Two large trials, Dapagliflozin and Prevention of Adverse Outcomes in Chronic Kidney Disease (DAPA-CKD) and the Study of Heart and Kidney Protection With Empagliflozin (EMPA-KIDNEY), have studied the impact of SGLT2i as a kidney-protective therapy in patients with CKD attributable to causes other than diabetes.^46^^,^^47^ These trials demonstrated that SGLT2 inhibition can substantially slow the progression of CKD in both diabetic and nondiabetic patients. Both trials included prespecified subgroup analyses of patients with IgAN as the cause of CKD and confirmed significant benefits in this population.^48^^,^^49^ In patients with IgAN who received SGLT2is, the mean rates of eGFR decline versus placebo were found to be –3.5 versus –4.7 ml/min per 1.73 m^2^ per year and –2.9 versus –4.0 ml/min per 1.73 m^2^ per year in the DAPA-CKD and EMPA-KIDNEY trials, respectively, supporting the use of these agents in patients with IgAN.^48^^,^^49^ SGLT2is are a recent addition to the management strategy of patients with IgAN, particularly in the context of reduced eGFR. SGLT2is are recommended in conjunction with RASis or a dual endothelin-angiotensin receptor antagonist in patients at risk of progressive loss of kidney function to reduce glomerular hyperfiltration and manage the consequences of nephron loss.^24^

### Endothelin antagonists

Endothelin-1 is produced by and acts on a variety of cell types within most organs. The kidney is a major source of endothelin-1, and each renal cell type expresses an endothelin receptor.^50^ Sparsentan, a dual endothelin-angiotensin receptor antagonist, was granted full US Food and Drug Administration approval in September 2024 to slow kidney function decline in adults with primary IgAN who are at risk for disease progression,^51^^,^^52^ and given standard European Medicines Agency marketing authorization in Europe in April 2025 for the treatment of adults with primary IgAN with a urine protein excretion ≥1.0 g/d (or urine protein-to-creatinine ratio ≥0.75 g/g). Continuous treatment with sparsentan for 110 weeks in patients with IgAN was associated with a 40% reduction in proteinuria and a chronic eGFR slope benefit compared with irbesartan, the active control.^14^ In the 2025 KDIGO guidelines, sparsentan is suggested as an alternative to RASis in patients who are at risk of progressive loss of kidney function.^24^ More information regarding this agent and the selective endothelin antagonist atrasentan can be found in the article titled “Approved therapies in the IgA nephropathy armamentarium: a summary of the evidence” by Norouzi and Lafayette^53^ of this supplement.

### Targeted immunomodulatory therapies

#### Nefecon (targeted-release formulation budesonide)

Nefecon, an oral, targeted-release capsule formulation of budesonide, was designed to deliver the active drug to the Peyer patches of the distal ileum to reduce mucosal Gd-IgA1 production in IgAN (see article “IgA nephropathy: an overview of the disease, its pathophysiology, and involvement of the gut-kidney axis” by Cheung and Mariani^54^ of this supplement for further information). It was the first agent to be approved specifically for the treatment of patients with IgAN and has received approval in the United States, China, European Union, and other territories.^55^, ^56^, ^57^, ^58^, ^59^, ^60^, ^61^, ^62^ Phase 3, multicenter, double-blind trial results of Nefecon, 16 mg/d, versus placebo treatment for 9 months (and a further 15 months off treatment) in patients with IgAN demonstrated statistically significant and clinically relevant benefits versus placebo. A difference in eGFR total slope of 2.95 ml/min per 1.73 m^2^ per year was shown at 24 months (95% confidence interval, 1.67–4.58 ml/min per 1.73 m^2^ per year; *P* < 0.0001), which represents 50% less deterioration of kidney function in Nefecon-treated patients compared with placebo-treated patients over the 2-year period.^12^ The incidence of infections was similar between the Nefecon group (35%) and the placebo group (31%) in contrast to systemic glucocorticoids (sGCs) that confer a higher risk of infection at high doses and are discussed later (see section titled Systemic glucocorticoids). The most common treatment-emergent adverse events reported with Nefecon were peripheral edema (17%), hypertension (12%), muscle spasms (12%), acne (11%), and headache (10%). The 2025 KDIGO guidelines suggest treatment with a 9-month course of Nefecon for patients who are at risk of progressive loss of kidney function as a means of managing the IgAN-specific immune drivers of nephron loss.^24^

More information regarding this agent can be found in the article titled “Approved therapies in the IgA nephropathy armamentarium: a summary of the evidence” by Norouzi and Lafayette^53^ in this supplement.

#### Iptacopan (complement-targeting therapy)

Iptacopan is an oral complement factor B inhibitor, and it has received accelerated approval in the United States for the reduction of proteinuria in patients with primary IgAN at risk of rapid disease progression, generally a urine protein-to-creatinine ratio ≥1.5 g/g. A phase 3, multicenter, double-blind, parallel-group study investigated treatment with iptacopan, 200 mg twice daily, versus placebo for 24 months in patients with IgAN. Interim analysis results showed that iptacopan was associated with a 38% reduction in urine protein-to-creatinine ratio versus placebo at month 9.^63^ Given the lack of approved medications to treat IgAN, the effects on proteinuria were sufficient to warrant accelerated approval, and full approval will require evidence of impact on eGFR in the full study population. More information regarding this agent can be found in the article “Approved therapies in the IgA nephropathy armamentarium: a summary of the evidence” by Norouzi and Lafayette^53^ in this supplement.

At present, iptacopan is the only complement-targeting agent to be approved for the treatment of IgAN. Other therapies in late-stage development include those targeting the alternative pathway, such as pegcetacoplan, a C3/C3b inhibitor approved for use in paroxysmal nocturnal hemoglobinuria, C3 glomerulonephropathy, and primary immune complex–mediated membranoproliferative glomerulonephritis.^64^^,^^65^ A phase 3 trial for ravulizumab (NCT06291376), a C5 inhibitor targeting the terminal pathway, is also enrolling participants at the time of writing.^64^^,^^66^ These and other therapies in development are discussed in the article “IgA nephropathy management: what does the future hold?” by Tang and Reich^67^ in this supplement.

### Other therapeutic interventions

#### Systemic glucocorticoids

sGCs have pleiotropic effects on the immune system, although there are currently no data on their effects on pathogenic forms of IgA (see the article titled “IgA nephropathy: an overview of the disease, its pathophysiology, and involvement of the gut-kidney axis” by Cheung and Mariani^54^ of this supplement for further information). A systematic literature review and meta-analysis assessing sGC therapy in patients with IgAN with >1 g/d proteinuria and normal kidney function, from 1966 to 2011, found 9 relevant trials including 536 patients.^68^ sGC therapy was found to be associated with a lower risk of kidney failure (relative risk, 0.32; *P* = 0.002) and a reduction in proteinuria (weighted mean difference of –0.46 g/d). High-dose and short-term therapy (prednisone >30 mg/d or high-dose pulse i.v. methylprednisolone with duration ≤1 year) showed significant kidney protection, whereas low-dose (prednisone <30 mg/d), long-term sGC use did not show a similar effect. Adverse event data were not consistently systematically collected in these studies, but a high risk of adverse events was associated with sGC treatment. However, these studies were all deemed “low” quality evidence and provided limited generalizability of the results.

Subsequent trials with larger sample sizes have been conducted to elucidate the potential benefits of sGC treatment in IgAN. The STOP-IgAN trial uniquely included German patients of European descent, and the Therapeutic Effects of Steroids in IgA Nephropathy Global (TESTING) trial recruited most patients from China.^16^^,^^17^^,^^69^^,^^70^

In the STOP-IgAN trial, following a 6-month run-in phase to optimize supportive care, patients with proteinuria >0.75 g/d were randomly assigned to receive supportive care alone (n = 80) or supportive care plus immunosuppressive therapy (sGC only if eGFR ≥60 ml/min per 1.73 m^2^ [n = 55] or sGC plus oral cyclophosphamide for 3 months, followed by azathioprine for 33 months, if eGFR <60 ml/min per 1.73 m^2^ [n = 27]) for 3 years.^16^ After this period, 6% versus 20% of patients in the supportive care alone and supportive care plus immunosuppressive groups, respectively, achieved full clinical proteinuria remission (*P* = 0.02; urine protein-to-creatinine ratio <0.2 and a decrease in the eGFR of <5 ml/min per 1.73 m^2^). No significant difference in the decline in eGFR between the 2 groups was noted (absolute eGFR change at 36 months was –4.7 ± 12.3 vs. –4.2 ± 14.1 ml/min per 1.73 m^2^ for patients in the supportive care alone and supportive care plus immunosuppressive groups, respectively; *P* = 0.32), although more adverse events were observed among patients who received immunosuppressive therapy. In a *post hoc* subgroup analysis designed to differentiate between the 2 immunosuppressive treatments, patients were more likely to achieve full clinical remission with sGC monotherapy versus supportive care, whereas patients treated with the combination of immunosuppressive treatments mentioned above achieved similar rates of clinical remission as supportive care.^71^ Finally, long-term follow-up STOP-IgAN data supported the conclusions of the initial study, highlighting no significant benefit to kidney function following the addition of immunosuppressive treatment to supportive care in the population studied. Adverse events occurred more often with systemic immunosuppression, including severe infections, impaired glucose tolerance, and weight gain.^16^^,^^17^

The TESTING-1 trial planned to assess oral methylprednisolone, 0.6 to 0.8 mg/kg per day, maximum 48 mg/d versus placebo for 2 months, with methylprednisolone to be tapered by 8 mg/d each month for a total of 6 to 8 months. The trial was terminated early because of a nearly 5 times higher risk of serious adverse effects.^70^ In the TESTING-2 trial, because of the excessive infections seen in the TESTING-1 trial, a reduced-dose protocol of 0.4 mg/kg per day (maximum, 32 mg/d) or placebo was included for 6 to 9 months with a 3.5-year follow-up in patients with IgAN (proteinuria ≥1 g/d and eGFR 20–120 ml/min per 1.73 m^2^).^69^ Methylprednisolone was found to significantly reduce the risk of the composite outcome of kidney function decline, kidney failure, or death due to kidney failure when compared with placebo, by a hazard ratio of 0.53 (95% confidence interval, 0.39–0.72; *P* < 0.001).^69^ However, incidence of adverse events was higher with methylprednisolone compared with placebo, and reduction in proteinuria was no longer evident 36 months after randomization.^69^^,^^70^

Treatment paradigms support the use of sGC to manage glomerular inflammation in patients with IgAN who are at risk of progressive kidney function loss, particularly given that Nefecon is currently unavailable in some countries. Treatment should be a limited course of reduced-dose sGC, as described in the reduced-dose protocol, and combined with *Pneumocystis jirovecii* pneumonia prophylaxis, once a full toxicity risk assessment has been completed.^24^

#### Tonsillectomy

The removal of tonsils is a process of surgically “debulking” the mucosa-associated lymphoid tissue and, in so doing, is thought to reduce the circulating levels of Gd-IgA1 seen in patients with IgAN by impeding the first step in the 4-hit IgAN pathogenesis pathway (see the article titled “IgA nephropathy: an overview of the disease, its pathophysiology, and involvement of the gut-kidney axis” by Cheung and Mariani^54^ in this supplement). Although tonsillectomy, alone or with pulsed GCs, has been associated with improved kidney survival and partial or complete remission (defined as a reduction in, or disappearance of, hematuria and/or proteinuria) in patients with IgAN, most of these results were from retrospective observational studies of patients based in Japan.^72^, ^73^, ^74^, ^75^ Hematuria as a purported marker of therapeutic response is referred to in the article “The expanding role of biomarkers in the management of IgA nephropathy” by Jain and Rizk^37^ in this supplement. Two small randomized controlled trials have been conducted, with one (N = 72) suggesting tonsillectomy combined with pulsed GC therapy has no beneficial effect over GC monotherapy in attenuating hematuria in patients with IgAN; however, an antiproteinuric effect was seen with combination therapy.^75^ The second study (N = 98) demonstrated a significantly shorter time to proteinuria and hematuria remission, greater cumulative remission rates for both hematuria and proteinuria, greater duration of remissions, and lower relapse rates with tonsillectomy plus standard of care versus standard care alone.^76^ The largest study of tonsillectomy in individuals outside of Pacific Asia was a comparison of outcomes of 61 patients who underwent tonsillectomy compared with a propensity-matched group within the European Validation Study of the Oxford Classification of IgAN (VALIGA) cohort.^77^ No significant correlation was found between tonsillectomy and kidney function decline in European patients with IgAN.^77^

On the basis of these data, the Japanese Society of Nephrology Guidelines recommend tonsillectomy for the treatment of patients with IgAN. The KDIGO guidelines do not recommend that tonsillectomy be routinely performed as a treatment for IgAN in non-Japanese patients.

#### Mycophenolate

Mycophenolate has been shown to cause immunosuppression via multiple pathways, such as inhibiting the proliferation of both B and T lymphocytes and suppressing the expression of some adhesion molecules, thereby decreasing the recruitment of lymphocytes and monocytes into sites of inflammation.^78^ Currently available evidence regarding the use of mycophenolate in patients with IgAN has been mixed. Results tend to differ in studies performed in cohorts of varying size and with varying disease severity.^79^, ^80^, ^81^, ^82^, ^83^, ^84^, ^85^, ^86^ For example, 2 larger randomized controlled trials (each ∼170 patients) conducted in China demonstrated benefit in lowering proteinuria and reducing eGFR decline when combined with supportive care or sGC.^79^, ^80^, ^81^, ^82^, ^83^ However, smaller randomized studies of mycophenolate demonstrated no meaningful impact on long-term kidney function,^79^^,^^80^^,^^84^ including 1 study that enrolled patients with more advanced CKD.^85^

In the absence of availability of novel, more effective therapies, mycophenolate remains a consideration as a treatment option, although evidence supporting its efficacy is mixed. The KDIGO guidelines have included consideration of mycophenolate as an option with reference to some positive results when studied in Chinese patients with IgAN as a possible GC-sparing agent.^24^

#### Hydroxychloroquine

Hydroxychloroquine (HCQ) is an established immunomodulator; it has immunologic and anti-inflammatory properties and has been used in managing autoimmune diseases.^87^, ^88^, ^89^ The value of HCQ in the management of IgAN in different racial groups and clinical disease severities is currently unclear. A systematic literature review of HCQ use in patients with IgAN included data describing 500 patients in total; there was a trend toward reduction in proteinuria in patients receiving HCQ, but no impact on eGFR was noted. Therefore, long-term studies with large patient populations, across different racial groups, are required to fully understand any potential benefits of HCQ in the management of IgAN.^90^

The processes of drug discovery, development, and approval are extremely costly. Consequently, novel agents treating IgAN are not universally accessible in all health care systems. Until these medications are globally accessible, more broadly available “traditional” medications will still be widely used to treat IgAN. Indeed, the 2025 KDIGO guidelines acknowledge that the cost or approval status of medications may limit their availability in resource-limited settings or countries, suggesting that reduced-dose sGCs be used in settings where Nefecon is not available.^24^ To assess the relative efficacy of these medications in IgAN, an important randomized adaptive platform clinical trial is underway in a large South Asian population (Alexander S, Raj Raj SS, Varughese S, et al. WCN25-3803: design of randomized embedded adaptive platform clinical trial in South Asian kidney biopsy-proven primary glomerular diseases: multi-center, multi-arm and multi-stage [abstract]. *Kidney Int Rep*. 2025;10:S772. Abstract 3803).^91^ The overarching hypothesis being tested is that use of broadly available drugs (oral prednisolone, enteric budesonide, HCQ, mycophenolate mofetil, or nonsteroidal mineralocorticoid antagonist) in addition to maximally tolerated RASi and SGLT2i can significantly improve 2-year kidney outcomes when compared with RASi and SGLT2i alone. This study is currently recruiting (NCT06676384) (Alexander S, Raj Raj SS, Varughese S, et al. WCN25-3803: design of randomized embedded adaptive platform clinical trial in South Asian kidney biopsy-proven primary glomerular diseases: multi-center, multi-arm and multi-stage [abstract]. *Kidney Int Rep*. 2025;10:S772. Abstract 3803).^91^

## Conclusions

IgAN is a chronic, progressive disease that is currently incurable, and many patients with IgAN ultimately progress to kidney failure.^1^^,^^12^^,^^17^ However, the course of the disease is clearly modifiable with treatment, particularly when interventions address the fundamental immunologic basis of kidney injury and nephron loss on a background of optimized supportive care. Recent trials demonstrate that with the use of these interventions, the rate of kidney function loss can be significantly slowed, if not stabilized, compared with supportive care alone.^12^^,^^14^

Our increased understanding of the pathogenesis of IgAN has provided many potential opportunities for intervention, which will hopefully lead to a more personalized approach when managing patients with IgAN. Indeed, the use of proteinuria and eGFR slope as surrogate end points has created opportunities to rapidly advance drug development research to find novel and more targeted treatments for IgAN.

**Figure 1:**
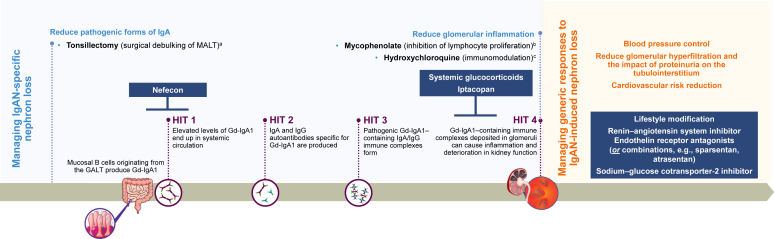
**Multimodal approach to managing patients with IgA nephropathy (IgAN).** The primary purported site of action of various therapies is noted in the figure, although some agents may have effects on multiple aspects of pathogenesis. ^a^The Japanese Society of Nephrology Guidelines recommend tonsillectomy for the treatment of patients with IgAN. However, updated Kidney Disease: Improving Global Outcomes guidelines do not recommend tonsillectomy as a treatment for IgAN in non-Japanese patients. ^b^The use of mycophenolate is reserved for Chinese patients with IgAN as a possible glucocorticoid-sparing agent. ^c^The value of hydroxychloroquine for the management of IgAN across different racial groups and clinical disease severities is currently unclear.^92^, ^93^, ^94^, ^95^, ^96^, ^97^, ^98^, ^99^ GALT, gut-associated lymphoid tissue; Gd-IgA1, galactose-deficient IgA1; MALT, mucosa-associated lymphoid tissue.

The unmet need for IgAN-specific, less toxic, immunomodulatory, targeted therapies is starting to be addressed by recently approved treatments, such as Nefecon and iptacopan. Given the increased number of agents being studied, and the changing treatment landscape toward more disease-targeted therapies with favorable toxicity profiles, the guidelines will also evolve rapidly. Indeed, the 2025 KDIGO guidelines recommend that treatments targeting both immune-mediated IgAN-specific drivers of glomerular injury (i.e., pathogenic Igs, immune complexes, and complement activations) and the downstream effects of nephron loss (hypertension, glomerular hyperfiltration, and the impact of proteinuria on the tubulointerstitium) should be considered simultaneously (Figure 1).^24^ The successful management of patients with IgAN may involve a multimodal approach, with therapies selected on the basis of individualized risk of progression and underlying pathophysiology. The incorporation of emerging biomarkers into treatment selection and modification could further facilitate personalized therapy across the spectrum of IgAN.

Great advances are being made in IgAN, including a deeper understanding of its pathogenesis, identification of pathologic and clinical biomarkers, and successful use of surrogate end points for clinical trials accepted by regulatory agencies. These advances have facilitated the development of multiple new therapeutic agents and are bringing to fruition the possibility of better long-term outcomes for patients with IgAN.

## Disclosure

This article is published as part of a supplement sponsored by Calliditas Therapeutics, an Asahi Kasei company.

PAC reports having received consulting fees from Novartis, Otsuka, Travere, Takeda, and Vera Therapeutics; and has served as a clinical trial investigator for Calliditas, Travere, Novartis, Vera Therapeutics, and Vertex Pharmaceuticals. HNR has received grant support from the Canadian Institutes of Health Research and the Kidney Foundation of Canada (from John and Leslie Pearson); has received research expenses (not personal fees) for clinical trials from Alexion, Calliditas Therapeutics, Novartis, and Omeros; reports consulting fees, honoraria, or travel support from Alexion, Biogen, Calliditas Therapeutics, Chinook, Novartis, Omeros, Otsuka, Pfizer, Travere Therapeutics, and Vera Therapeutics; has served on advisory boards and steering committees for Alexion, Chinook, Novartis, Omeros, Otsuka, Pfizer, and Travere Therapeutics; has been an investigator for Alexion, Alnylam Pharmaceuticals, Calliditas Therapeutics, ChemoCentryx, Chinook, Novartis, Omeros, and Pfizer; and is director of the Glomerulonephritis Fellowship, supported by the Louise Fast Foundation and Otsuka Canada. She served as a coauthor of the 2025 Kidney Disease: Improving Global Outcomes Clinical Practice Guideline for the Management of Immunoglobulin A Nephropathy and Immunoglobulin A Vasculitis.
